# Assessment of Immunization Status in the Slums of Surat by 15 Clusters Multi Indicators Cluster Survey Technique

**DOI:** 10.4103/0970-0218.51222

**Published:** 2009-04

**Authors:** Rashmi Sharma, Vikas K Desai, Abhay Kavishvar

**Affiliations:** Department of Community Medicine, Kesar SAL Medical College & Research Institute, Ahmadabad, India; 1 Department of Community Medicine, Government Medical College, Surat, India

**Keywords:** 12-23 months, immunization status, MICS, slums

## Abstract

**Research Question:**

What is the immunization status of children in the slums of Surat and what changes has it undergone in recent times?

**Objective:**

To assess the immunization status of children between the ages of 12 and 23 months in the slums of Surat and to compare it with the MICS from previous years.

**Study Design:**

This was a community-based cross-sectional study conducted in 15 clusters.

**Settings:**

15 urban slums selected out of a total of 299 slums using the cluster sampling method.

**Study Tool:**

The Multi Indicator Cluster Sampling (MICS) method was used for sample selection and the proforma designed by UNICEF was used as a study tool.

**Statistical Analysis:**

Simple proportions and a Chi-square test.

**Results:**

Only 25% of the children between the ages of 12 and 23 months were fully immunized; coverage was highest for BCG (75%) and lowest for measles (29.9%). As far as the dropout rate is concerned, it was 60.2%, 31.9%, and 31.5% for BCG to measles, DPT_1_ to DPT_3_, and OPV_1_ to OPV_3_, respectively. Vitamin A was taken by only 28.9% of the subjects. Between the two, female children were more disadvantaged in terms of vaccination. When compared with the coverage of 1997 and 1998, the current coverage is poor, more so in relation to DPT and OPV.

## Introduction

The current scenario depicts that immunization coverage has been steadily increasing but the average levels remain far less than desired. Still only 44% of infants in India are fully immunized (NFHS III), which is much less than the desired goal of achieving 85% coverage.(1) Because of increased accessibility of health care services in both urban and rural areas, an increase was expected in the utilization of the services, however, studies reveal low utilization of health care services including MCH services(2) by different segments of the society. Slums are high-risk areas leading to a high rate of disease transmission and about 25% of the Indian urban poor currently live in slums. Maternal and child health indicators among slum people show that their health is 2-3 times worse than in urban areas.(3) This study was formulated against this background with an objective of assessing the immunization coverage in the slums of Surat, as about 40% of the population of Surat resides in slums. An additional objective was to find out the change in the scenario in comparison with previous years. Studies focusing on slums are essential in order to achieve the desired vaccination coverage in slums (unique ethnic and sociological characteristics) and extra efforts are needed for the IEC organization and mobilization of the parents.

The MICS technique proposed by the World Health Organization (WHO) with 15 or 30 clusters is a popular method for rapid assessment of health service coverage and their impact evaluation. A rapid assessment of immunization is best achieved by assessing the primary vaccination done during infancy by administering 4 vaccines (BCG, DPT, OPV, and measles) through 5 properly spaced contacts. Since the MICS survey is conducted among children between 12 - 23 months old for their immunization experience during infancy (0 - 11 months), it actually reflects the vaccination performance of the preceding year.

## Materials and Methods

Surat is the second largest city (next to Ahmadabad) of Gujarat with a population of 3.5 million residing in the area of 112 square kilometers (at the time of study). The city has a large number of migrants both interstate (from Maharashtra, Orissa, Rajasthan, UP, and Bihar) as well as intrastate (from Saurashtra and North Gujarat). More than 40% of the city population lives in slums characterized by poor sanitation, poverty, overcrowding, congested living, and a lack of personal hygiene. Most of the slum dwellers are engaged in unskilled jobs or semi-skilled jobs such as diamond cutting, polishing, and organized/semi/unorganized textile (power) looms. As far as the health facilities are concerned, the city has 2 teaching hospitals and a network of 24 urban health centers (UHC) run by the Municipal Corporation. There is a network of 43 female health workers attached to the UHC and 2 integrated child development scheme projects catering mainly to the slum population (population under study) and also facilitating the vaccination services. In addition, there are several big to medium-sized private hospitals and practicing care providers.

Two MICS surveys^4^ have been undertaken in the slums of Surat in 1997 and 1998 to monitor the immunization services for the years of 1996 and 1997, respectively. The present communication deals with the findings of the MICS survey undertaken in July - September 2000 that actually reflects the immunization status of the preceding year i.e., 1999.

The study population comprised of the people residing in 299 slums located within the areas under the Surat Municipal Corporation (SMC). The study sample included 15 clusters from all 299 slums selected through the cluster sampling method as proposed by WHO. A total of three teams (each comprising of 1 faculty member and 2 health workers) did the survey using a structured and pre-tested questionnaire designed by UNICEF. Team members received practical training and also had an extensive discussion about it.

### Selection of study clusters:

A list of all slums (299) with a population of 433,600 was procured. A cluster interval (28,906) was obtained by dividing the total population by 15 (number of clusters). A random number less than the cluster interval (24,894) were generated with the help of a currency note. The cluster, which represented this number, was picked up as the first cluster and subsequent clusters were selected by adding the cluster interval of 28,906. Thus, 15 clusters were selected on the basis of systematic random sampling from the probability of the cluster selection based on the population size of the cluster. In each cluster, households were studied in a sequence until a total of 20 children in the age group of 12 -23 months were covered. Data thus gathered was analyzed by using the EPI-INFO Version 6 package and simple proportions were calculated and statistical tests of significance were applied wherever found necessary.

## Observations

A total of 2,145 families with 11,336 subjects were studied from 15 clusters. As per the sample design, a total of 300 children (20 children per cluster) between the ages of 12 and 23 months old were to be studied. The population in the 15 selected clusters includes 1,371 preschool children, 2,161 children of school going age, 2,251women in the reproductive age group, 240 mothers who delivered in the last 12 months, and 294 children between 12 and 23 months old [Table 1].

**Table 1:** Demographic characteristics of study sample

Characteristics	Number
Households	2145
Population	11336
Children under 5 years old	1371
Children between 5-14 years old	2161
Women between 15-49 years old	2251
Mother who delivered within last 12 months	240
Children between 12-23 months	294

### Immunization status:

Actually, 300 children between the ages of 12 - 23 months were covered; however, the full information was not available for 6 children so subsequent analysis has been done in 294 children only. The observations among these children are listed in this article. Immunization cards were available for 79 (26.8%) children; for the remaining children, parental recall was relied up on.

Among individual vaccines, coverage was highest for BCG (75.1%) and lowest for measles (29.9%). Coverage for DPT_3_ and OPV_3_ was almost the same (48.6% and 47.9 %). Only 85 (28.9 %) received Vitamin A supplements at the time of measles vaccination. A consistent decline in coverage rate from the first to third dose was observed in DPT and OPV. DPT and OPV dropout rates from the first to third dose were 31.9% and 31.5%, respectively. The dropout rate for measles compared with BCG and DPT_3_ were 60.2% and 57.5%, respectively. Only 74 (25.1%) were fully immunized. The rest were either partially immunized (51.7 %) or not immunized at all (23.1 %).

When compared between two genders [Table 2], the proportion of fully immunized children was higher in females (27.3%) than in males (23.4%), however the difference was statistically not significant (×^2^ = 0.58, *P* > 0.05). Coverage for DPT_3_, OPV_3_, and BCG were high for male children. All differences were significant (×^2^ = 4.75, *P* < 0.05 for DPT; ×^2^ = 3.03, *P* > 0.05 for OPV; and ×^2^ =7.74; *P* < 0.005 for BCG). However, the coverage was higher for Measles in females (32.8 %) than in males (27.8 %) but the difference was not significant (×^2^ = 0. 98 *P* > 0.05). Dropout rates for OPV were almost the same in male and female children (31.5% and 31.6%). For DPT, the dropout rate was higher in females (32%) than in males (28%); the differences here were statistically not significant (× = 3.75, *P* > 0.05). Coverage for Vitamin A along with measles vaccination was more in male children but statistically the difference was not significant (×^2^ = 0.27, *P* > 0.05). The Measles dropout rate in relation to BCG and first dose DPT was higher in male children than in female children but statistically differences were not significant (×^2^ = 0.90, *P* > 0.05 for BCG to Measles and ×^2^ = 9.91, *P* < 0.001 for DPT_1_ to Measles).

**Table 2:** Immunization coverage and dropout rates among study subjects

Vaccination status	Total		Male		Female	
			
	No	%	No	%	No	%
Total children (N)	294		166		128	
Fully immunized	74	25.1	39	23.4	35	27.3
Partially immunized	152	51.7	101	60.8	51	39.8
Not immunized	68	23.1	26	15.6	42	32.8
BCG	221	75.1	135	81.3	86	67.1
DPT_1_	207	70.4	129	77.7	78	60.9
DPT_2_	179	60.8	110	66.2	69	53.9
DPT_3_	143	48.6	90	54.2	53	41.4
OPV_1_	206	70	127	76.5	79	61.7
OPV_2_	179	60.8	110	66.2	69	53.9
OPV_3_	141	47.9	87	52.4	54	42.1
Measles	88	29.9	46	27.8	42	32.8
Vitamin A (First dose)	85	28.9	46	27.7	39	30.4
Drop out rates (%)						
DPT (I to III)	31.9		28		32	
OPV (I to III)	31.5		31.5		31.6	
BCG to Measles	60.2		65.8		51.1	
DPT_1_ to Measles	57.5		64.2		46.1	

## Discussion

The MICS 1997 conducted in 15 clusters served as a baseline for Surat. In the MICS 1998, 300 children (12 - 23 months old) were assessed for immunization coverage and the coverage rates were 75%, 76.3%, and 51.3% for BCG, DPT_3_, and OPV_3_, respectively - higher than the present study.(4) Surat Municipal Corporation (1999) shows 100% coverage and nil dropout rates, which shows discrepancies between the reported coverage by providers in terms of target assigned to them and the coverage obtained by external evaluation done through such exercises. A study conducted in 1987 also in the slums of Surat(5) observed low coverage for BCG (43.2%), OPV_3_ (52.1%), and DPT_3_ (37.1%). A report from a remote tribal district of Gujarat (1997)(6) found 39% of the children were fully immunized, which is higher than this study. Another study (1991) concluded more a pronounced dropout rate for measles than for OPV and DPT.(7) Measles coverage of 30% in our study was worse than 49.8% reported by Desai, *et al.* (2003) in the same area in the children of the same age group.(8) It was little less (48.3%) among children under 5 years old, which can be attributed to a longer recall period. Such a low coverage for measles vaccine in our study can be because of various reasons, such as (i) disease not considered a serious disease by parents, health workers, and even some doctors, (ii) adverse reactions leading to a few unfortunate deaths after improper measles vaccine (mostly a technical error) are widely publicized leading to restrictive directives (vaccine to be administered only in the presence of doctor), (iii) Health workers accustomed to shortages and accountability are reluctant to open 10 dose vials for 1 or 2 eligible infants, (iv) current sickness/ history of sickness of child lead service provider to withhold the vaccine (v) mothers give history of measles and fail to vaccinate their children (mother may be mistaken). Malnourished children at 9 months old appear younger, so health workers and even doctors may decide not to vaccinate. Parents/ health workers forget about the vaccine due to 5-6 months interval between DPT_3_ and OPV_3_ and measles vaccination.(9) In our study, most of those who received a measles vaccine (85 out of 88) also received a first dose of vitamin A. Therefore, any attempt to improve measles vaccinations will also improve the coverage of Vitamin A.

Pulse Polio Immunization since 1995 has taken a toll on all other vaccines and that is the reason for poor coverage in this study when compared with those of 1997 and 1998. Exclusive emphasis on polio eradication and ongoing frequent rounds of pulse polio immunizations (PPI) effect the intake of other vaccines as the entire IEC activity focuses only on polio and not other vaccine preventable diseases (VPD). Health workers too are busy throughout the year in several rounds of pulse polio immunization leaving little time for routine vaccination. It has affected all vaccines except BCG, which is given at the time of birth or during the first contact with health functionaries. Single dose administrations such as BCG requires little parent's motivation.

**Figure 1:**
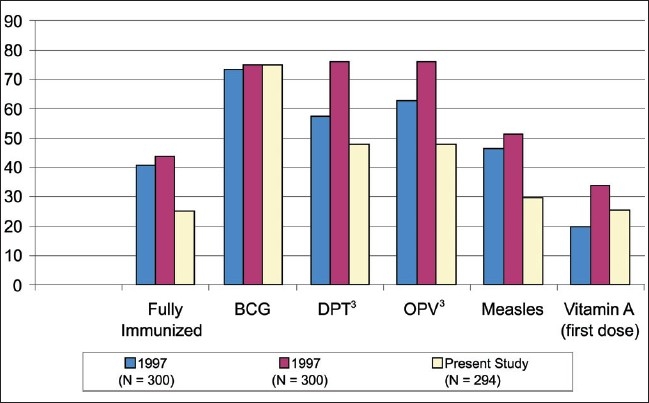
Percent vaccination coverage and its comparison with previous years

The proportion of fully vaccinated children in our study was 25.5%, which is less when compared with other studies either from the same area (1997 and 1998 - Figure 1) or otherwise. Compilation reports (1999) in major towns of Gujarat (Ahmadabad, Rajkot, and Jamnagar)(10) revealed overall vaccination coverage between 46.7% and 58.9% with Baroda reporting even higher coverage (78.6%). Declining vaccination rates for DPT, OPV, and Measles shall be a matter of concern to all of us.

National statistics (NFHS III)(1) reflects fully immunized children as 44% with individual coverage for BCG, DPT, polio, and Measles as 78%, 55%, 78%, and 59%, respectively, which is comparatively high but is nowhere near the target of 85% of complete immunization. Reasons for non immunization should not only be identified but appropriately addressed by effective communication efforts.(1) Specific IEC should target the parents focusing to differentiate between routine vaccinations and polio campaigns. In simpler terms, “6-8 doses of OPV in a year are no substitute for DPT and Measles”.

## Conclusion

Utilization of immunization services in the slums of Surat is low compared with other studies probably due to the fact the study area is a slum with its unique characteristics (floating population, overcrowding and poor sanitation and personal hygiene). Slums do have more morbidity withholding vaccinations by paramedics, as well as there no one being at home to take the child to health services for vaccinations. Coverage for fully immunized children was primarily low due to measles. It can be advised that some health care packages under maternal and child health (MCH), such as family planning counseling, iron, folic acid or vitamin A supplementation, or the provision of iodized salt can be given to attract parents especially to sustain contact for the time between DPT_3_ and Measles vaccinations and to hold the parents attention during non immunization periods and also to contribute toward the health status of the mother and children.

## Recommendations

Renewed interest should be developed both in local health functionaries and beneficiaries to accelerate the optimization of immunization services.

The RCH program for immunization should revise its strategy to increase the utilization of services by all segments of the population.

Improvement should focus on bottlenecks by reducing the dropout rate from DPT_2_ /OPV_2_ to DPT_3/_OPV_3_ and improving coverage of measles (and also Vitamin A).

The remaining deficiency may be overcome by generating awareness among the community by holding mother's meetings and extensive IEC programs, inviting opinions and suggestions from them, and enhancing community participation.
